# Oxygen-Independent Antimicrobial Photoinactivation: Type III Photochemical Mechanism?

**DOI:** 10.3390/antibiotics9020053

**Published:** 2020-01-31

**Authors:** Michael R Hamblin, Heidi Abrahamse

**Affiliations:** 1 Wellman Center for Photomedicine, Massachusetts General Hospital, Boston, MA 02114, USA; 2 Department of Dermatology, Harvard Medical School, Boston, MA 02115, USA; 3 Laser Research Centre, Faculty of Health Science, University of Johannesburg, Doornfontein 2028, South Africa; habrahamse@uj.ac.za

**Keywords:** antimicrobial photodynamic inactivation, oxygen-independent photoinactivation, bacteria, psoralens, tetracyclines, potassium iodide, sodium azide

## Abstract

Since the early work of the 1900s it has been axiomatic that photodynamic action requires the presence of sufficient ambient oxygen. The Type I photochemical pathway involves electron transfer reactions leading to the production of reactive oxygen species (superoxide, hydrogen peroxide, and hydroxyl radicals), while the Type II pathway involves energy transfer from the PS (photosensitizer) triplet state, leading to production of reactive singlet oxygen. The purpose of the present review is to highlight the possibility of oxygen-independent photoinactivation leading to the killing of pathogenic bacteria, which may be termed the “Type III photochemical pathway”. Psoralens can be photoactivated by ultraviolet A (UVA) light to produce DNA monoadducts and inter-strand cross-links that kill bacteria and may actually be more effective in the absence of oxygen. Tetracyclines can function as light-activated antibiotics, working by a mixture of oxygen-dependent and oxygen independent pathways. Again, covalent adducts may be formed in bacterial ribosomes. Antimicrobial photodynamic inactivation can be potentiated by addition of several different inorganic salts, and in the case of potassium iodide and sodium azide, bacterial killing can be achieved in the absence of oxygen. The proposed mechanism involves photoinduced electron transfer that produces reactive inorganic radicals. These new approaches might be useful to treat anaerobic infections or infections in hypoxic tissue.

## 1. Introduction

The ever-growing development of antibiotic resistance amongst pathogenic bacteria and other microorganisms has led to the prediction of the “end of the antibiotic era” [1]. The fear has been voiced that we could return to an age when even minor injuries could prove fatal when infections set in, and eventually lead to sepsis. The O’Neill report [2] caused widespread consternation with its prediction of 300 million extra deaths and a cost of $100 trillion by 2050 if nothing were done to halt the spread of antibiotic resistance. There has therefore been a worldwide effort to discover alternative antimicrobial technologies, which will kill multi-drug resistant microbial species, as well as not cause the development of resistance themselves. One of the leading candidates for this new antimicrobial approach is antimicrobial photodynamic inactivation (aPDI).

## 2. Antimicrobial Photodynamic Inactivation

Photodynamic therapy was discovered over 100 years ago, when Oscar Raab (a student of Hermann von Tappeiner) in Munich, Germany accidentally observed the killing of a type of microorganism called infusoria, in the simultaneous presence of the fluorescent dye, acridine orange, and light delivered by a lightning in a thunderstorm [3]. A few years later they discovered that the presence of ambient oxygen was also necessary for the killing reaction to occur, and coined the term “photodynamic” to describe it [4]. For many years afterwards this effect was mainly studied as a type of cancer treatment called “photodynamic therapy” (PDT) [5]. Several photosensitizers (PS) were investigated for PDT, including hematoporphyrin derivative or Photofrin, which was championed by Thomas J Dougherty from Roswell Park in Buffalo, USA [6]. Around the 1990s, PDT started to be studied as an antimicrobial technique, when it was discovered that Gram-negative bacteria that had been previously thought to be resistant to PDT [7,8], could be efficiently killed by using PS with cationic charges [9] or other modifications to the procedure [10,11].

Since those days, there has been a large number of publications investigating antimicrobial photodynamic inactivation (aPDI), a term generally preferred to aPDT for in vitro killing studies of microbial cells or viruses. A large range of PS have been described for aPDI based on a wide variety of basic backbones, including porphyrins, chlorins, bacteriochlorins, phthalocyanines, phenothiaziniums, xanthenes, BODIPYs, and fullerenes to name just a few [12]. In recent years efforts have been made to improve the effectiveness of aPDI using approaches such as conjugation of PS to cationic polymers [13], use of sophisticated nanoparticle-based approaches [14], and potentiation of microbial killing by addition of inorganic salts [15].

Antimicrobial PDT has been demonstrated to be a successful technology in a variety of different laboratory animal models of bacterial and fungal infection [16], in clinical veterinary practice [17], and in human clinical trials in dentistry [18] and chronic wounds [19].

## 3. Type I and Type II Photochemical Mechanisms

**Figure 1:**
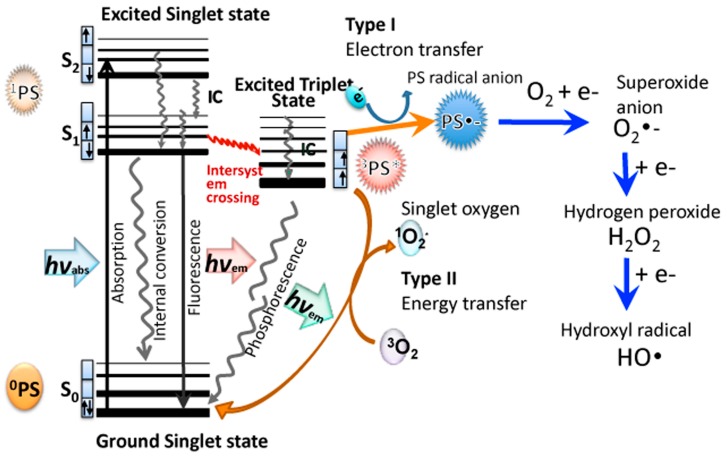
**Jablonski diagram.** Illustrating Type I electron transfer pathway producing superoxide, hydrogen peroxide, and hydroxyl radicals, and Type II energy transfer pathway producing singlet oxygen.

Christopher Foote was one of the first to distinguish between the two photochemical mechanisms involved in PDT, called Type I and Type II [20]. A recent review has elaborated on these two pathways [21]. When the PS absorbs a photon of light, the electron in the HOMO (highest occupied molecular orbital) is excited into the LUMO (lowest excited molecular orbital), initially producing the first excited singlet state (^1^PS*). The ^1^PS* usually has a very short lifetime (few nsec) because it rapidly loses energy by radiative (fluorescence) or non-radiative decay pathways (internal conversion). However, a certain fraction of the ^1^PS* molecules can undergo an intersystem crossing process, involving a spin-flip of the outermost electron to the excited triplet state (^3^PS*). Figure 1 shows a Jablonski diagram illustrating these mechanisms. The ^3^PS* has a much longer lifetime (µsec) than ^1^PS*, which enables it to survive long enough to undergo physical or chemical reactions. The electronic selection rules allow interactions between triplets and other triplets, while interactions between triplets and singlets are spin-forbidden. One of the few molecules that exists as an electronic triplet in its natural ground state is dioxygen (^3^O_2_). This means that ambient oxygen is able to react readily with the triplet PS. One important reaction that can occur is a physical energy transfer process called Type II, which produces the ground state singlet PS molecule ^1^PS^0^, and the excited state singlet oxygen molecule ^1^O_2_*. Singlet oxygen is a reactive oxidizing agent that can typically add to double bonds (ene-type addition) or add across aromatic rings to form endoperoxides. The singlet oxygen has a short lifetime because it can be physically quenched by collision, or chemically quenched by reacting with surrounding molecules. Singlet oxygen lifetimes are much longer in lipophilic environments than they are in aqueous surroundings.

On the other hand, the ^3^PS* can also undergo an electron transfer reaction (called the Type I pathway) in the presence of an electron donor to produce the PS radical anion, PS^-•^. PS molecules that have lower redox potentials (in their ground state) are more likely to be able to accept an electron. The PS^-•^ can then undergo an electron transfer reaction with ground state oxygen to produce superoxide radical anion O_2_^-•^. There can then follow a second 1-electron reduction of superoxide to produce hydrogen peroxide (H_2_O_2_) and a third 1-electron reduction to produce hydroxyl radical (HO^•^). Moreover, Fenton chemistry involving Fe(II) species may also increase the amount of HO^•^ produced ref. These three different reactive oxygen species (ROS) (O_2_^-•^, H_2_O_2,_ HO^•^) are collectively termed Type I ROS.

Although all the four different ROS produced during aPDI (^1^O_2_*, O_2_^-•^, H_2_O_2,_ HO^•^) have been shown to kill microbial cells, ^1^O_2_* and HO^•^ are the most powerful microbicidal agents, but because they are so reactive they have only a short diffusion distance, and therefore can only kill pathogens located very close to where they are produced. For this reason, binding of the PS to the bacterial cell, and penetration through the outer cell wall to reach the plasma membrane or the cytoplasm, is an important advantage. Relatively small PS molecules with cationic charges and also an asymmetric structure are the most potent molecules for broad-spectrum microbial killing [22,23].

## 4. Infections in Hypoxic and Anaerobic Tissue Environments

Some pathogenic bacteria are obligate aerobes (Pseudomonas aeruginosa, Neisseria meningitides, Mycobacterium tuberculosis), while others are obligate anaerobes (Clostridium spp, Prevotella spp, Porphyromonas gingivalis, Propionibacterium acnes). However, the majority of pathogenic bacteria are classified as facultative anaerobes. Bacteria carry out aerobic respiration at O_2_ concentrations between atmospheric levels (21% v/v) down to 0.5%. Between 0.5% and 0.1% the process shifts to anaerobic respiration, while below 0.1% O_2_ v/v, the process of fermentation takes place [24]. Anaerobic infections occur in the gut, mouth, head and neck region, and in a variety of abscesses situated in different parts of the body. Because bacterial killing by neutrophils is an important aspect of host defense, and because neutrophils require sufficient oxygen to carry out this role effectively, bacterial infections are more likely to occur in hypoxic tissue. Many infections also arise in damaged or wounded tissue, because the protective barrier to the outside environment has been breached, and in injured tissue the disrupted blood supply restricts the supply of oxygen, as well as limiting the access of systemically administered antibiotics. It was shown that the wound tissue oxygen tension predicted the risk of surgical site infections in post-operative patients [25]. Hyperbaric oxygen therapy has been used as a treatment for a variety of different infections, but the mechanisms are not completely understood [26].

## 5. Type III Photochemical Mechanism

It has often been stated that high oxygen partial pressures encourage a Type II photochemical mechanism, while lower pO2 values encourage a Type I photochemical mechanism [27,28], although hard evidence of this assertion is somewhat elusive. Nevertheless, it is clear that for the vast majority of PS and experimental conditions, a sufficient concentration of oxygen is critical for the optimum photodynamic action. Although the use of the term “photodynamic” implies by definition the involvement of oxygen, we would like to propose that oxygen-independent photoinactivation of microbial species should be called the “Type III photochemical pathway”.

## 6. Psoralens

**Figure 2:**
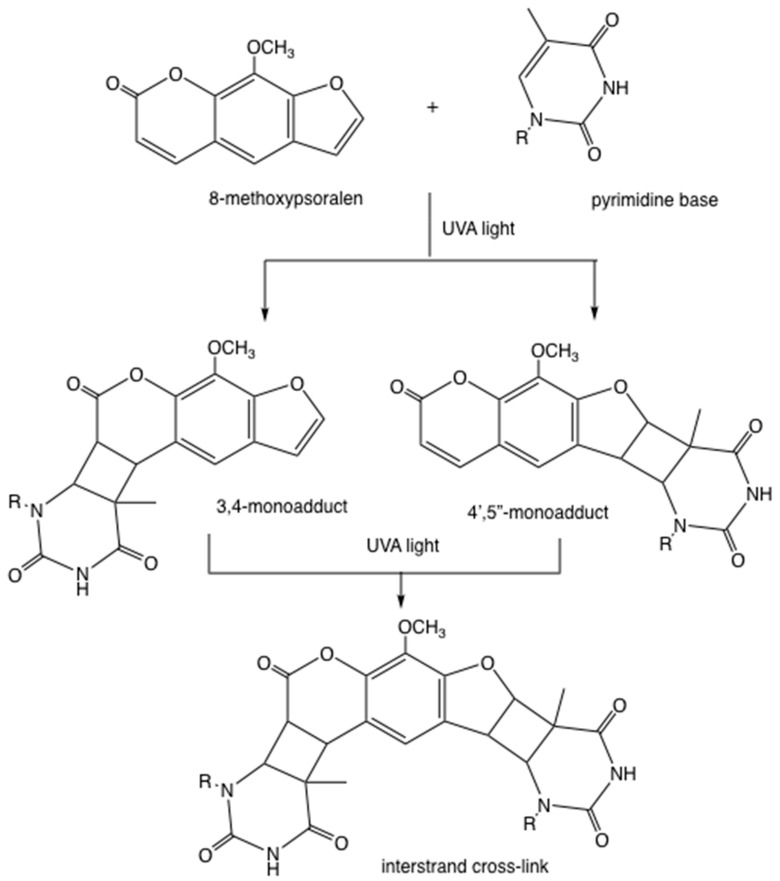
**DNA cross-linking by photoactivated psoralens.** Example shown is 8-methoxypsoralen forming either a 3,4-mono-adduct or else a 4’,5’-monoadduct with a thymine base. These mono-adducts can absorb a second ultraviolet-A (UVA) photon and form an inter-strand cross-link with a second thymine base in the opposite strand.

Psoralen is the parent compound of a group of linear furanocoumarins, which occur naturally in a variety of plants and foodstuffs. The use of light-activated psoralens as a medical treatment dates back thousands of years to ancient Egypt and India, where they were used to treat vitiligo (a disfiguring skin disease) [29]. Preparations were made from *Psoralea coryrifolia* or *Ammi majus*, and either applied topically or consumed orally followed by exposure of the skin to strong sunlight. In 1948, el Mofty in Egypt reported the use of purified 8-methoxypsoralen (8-MOP, Figure 2) and sunlight to treat vitiligo (leukoderma) [30], and in 1974 Parrish and Fitzpatrick used 8-MOP plus UVA illumination from a new high power fluorescent tube for treatment of psoriasis (an autoimmune skin disease) [31]. This treatment became known as psoralen-UVA (PUVA) and was widely used throughout the world, until it was realized that the treatment itself carried a risk of inducing skin cancer [32].

The mechanism of action of PUVA is different from PDT, although both techniques employ a photosensitizing drug activated by light. Psoralen molecules intercalate into the double helix of DNA, and upon photoactivation, the pyran ring of the psoralen can either form a 3,4-mono-adduct by undergoing a 2+2 cycloaddition reaction with thymine (or uracil in RNA), or less likely, the furan ring can form a different 4’,5’-monoadduct (Figure 2). In some cases, these mono-adducts can absorb a second UVA photon and form an inter-strand cross-link with a second thymine base (Figure 2). Neither of these reactions require the involvement of oxygen molecules. 

As long ago as 1959, Oginsky et al explored many of the determinants of bacterial photoinactivation mediated by photoactivated psoralens [33]. They compared the killing of *S. aureus* and *E. coli* using either methylene blue excited by visible light or 8-methoxy psoralen (8-MOP) excited by UVA light. Interestingly, while the number of logs of killing of *S. aureus* that were achieved using MB + white light was sharply reduced (5 logs less) when the oxygen was replaced with nitrogen, in the case of 8-MOP + UVA the logs of killing were actually increased (from 4 logs to 5 logs) by replacing oxygen with nitrogen. A similar trend was observed for *E. coli*, although the absolute number of logs of killing was much lower. The observation of the increased bacterial killing in the absence of oxygen by photoactivated psoralens was confirmed by Bianchi et al [34], who were looking at photomutagenesis of bacteria, but could not measure it in the absence of oxygen because the bacteria were totally eradicated.

**Figure 3:**
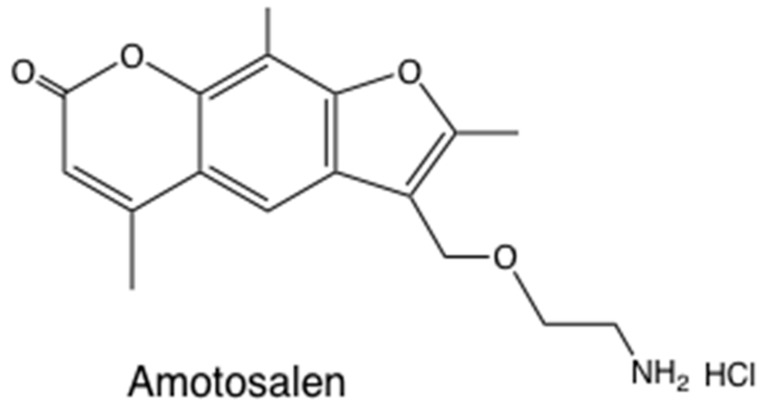
**Amotosalen structure.** 3-(2-aminoethoxymethyl)-2,5,9-trimethylfuro[3,2-g] chromen-7-one HCl.

The clinical applications of the antimicrobial activity of photoactivated psoralens have largely centered on sterilization of blood products (plasma and platelet concentrates) [35]. Both viruses [36] and bacteria [37] can be efficiently killed. Amotosalen is a synthetic aminopsoralen (Figure 3) that has been developed by Cerus Corp as the Intercept® system for pathogen inactivation [38]. Platelet concentrates were treated with amotosalen (40 µM) plus 3.9 J/cm^2^ UVA light [39]. 

## 7. Tetracyclines

The discovery of the tetracyclines (TCs) was a major step forward in the search for naturally occurring antibiotics [40]. Starting with aureomycin, and progressing through terramycin, tetracycline, doxycycline, demeclocycline, and minocycline, these antibiotics saved countless lives throughout the world [41]. The mechanism of action of tetracyclines involves binding to bacterial ribosomes (30S ribosomal subunit), preventing tRNAs from binding to the aminoacyl–mRNA complex, and thereby inhibiting protein synthesis [42,43]. This ribosomal binding is reversible, which is why tetracylines are considered to be bacteriostatic in nature, rather than bactericidal [44].

Fairly soon after the clinical use of tetracyclines became established, reports began to emerge concerning a worrying side-effect observed in some patients, namely skin photosensitivity [45] and photo-onycholysis [46]. Tetracyclines (and in particular demeclocycline) are now accepted to be important causes of drug-induced photosensitivity reactions [47]. Most tetracyclines have absorption peaks in the UVA region of the spectrum, while in some compounds the peak extends into the blue region. It has also been proposed that some tetracycline photo-products can absorb at longer visible wavelengths [48]. Hasan and Khan investigated the photochemical mechanism responsible for the photosensitivity of tetracyclines [49]. They concluded that when TCs were activated by UVA light, they produced singlet oxygen by a type II photochemical mechanism, and this was responsible for the skin photosensitivity. The singlet oxygen quantum yields were measured to be: demeclocycline, 0.08; tetracycline, 0.05; minocycline, 0.00. The lack of singlet oxygen produced by minocycline was in agreement with the fact that clinical photosensitivity has not been reported for this particular antibiotic.

Our laboratory has recently studied whether tetracyclines could be used as light-activated antibiotics [15]. We first compared four compounds: tetracycline, doxycycline, demeclocycline, and minocycline [50]. Doxycycline was excited by UVA light (365 nm) while demeclocycline was activated by blue light (415 nm). Both compounds were able to completely eradicate Gram-positive (MRSA, methicillin-resistant *Staphylococcus aureus*) and Gram-negative (*Escherichia coli*) bacteria (>6 log(10) steps of killing) at concentrations (10–50 μM) and fluences (10–20 J/cm^2^). The killing was only slightly reduced after a “wash” step, showing the tetracyclines bound tightly to the bacterial cells. Tetracyclines plus light killed bacteria incubated in rich growth medium, which was in sharp contrast with methylene blue (MB) activated by red light. While photoactivated MB effectively eradicated bacteria incubated in phosphate buffered saline, this activity was completely lost in growth medium. When ~3 logs of bacteria were killed with demeclocycline plus light, and the surviving cells were added to growth medium, further bacterial killing was observed, while when the same experiment was carried out with MB we observed complete regrowth. Minimal inhibitory concentration (MIC) studies were carried out either in the dark or continuously exposed to 0.5mW/cm^2^ blue light. Up to three extra steps (8-fold) increased antibiotic activity was found with light compared to dark, using MRSA and tetracycline-resistant strains of *E. coli*. We also showed that in a mouse model of a skin abrasion infected by bioluminescent *E. coli,* topical application of DMCT (demeclocycline) followed by illumination with blue light produced a light dose-dependent loss of bioluminescence, not seen with blue light or DMCT alone. Moreover, monitoring of the bioluminescence signal over the succeeding 5 days showed that the DMCT + blue light prevented any bacterial regrowth. This prevention of regrowth was proposed to be due to the presence of remaining unactivated DMCT acting as a bacteriostatic antibiotic within the wound [51].

**Figure 4:**
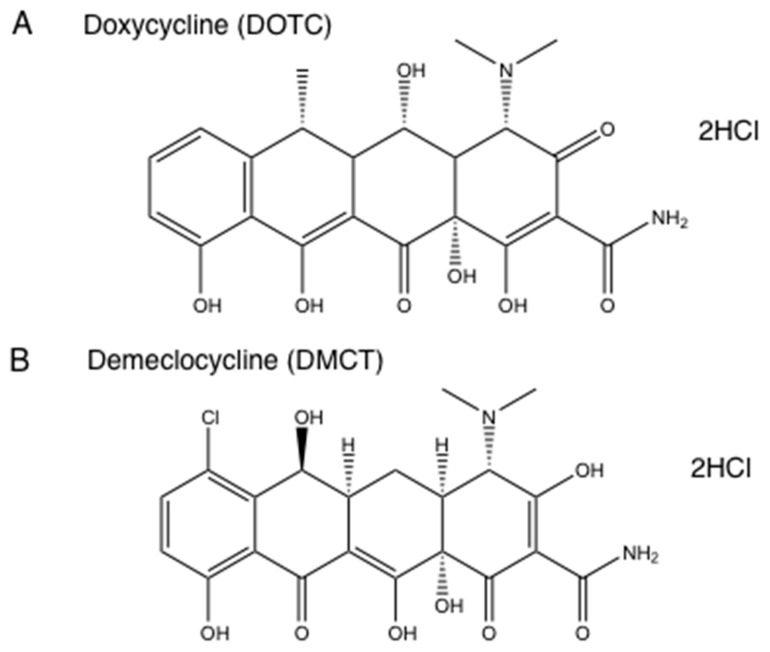
**Tetracycline structures.** (**A**) Doxycycline hydrochloride (DOTC); (**B**) Demeclocycline hydrochloride (DMCT).

**Figure 5:**
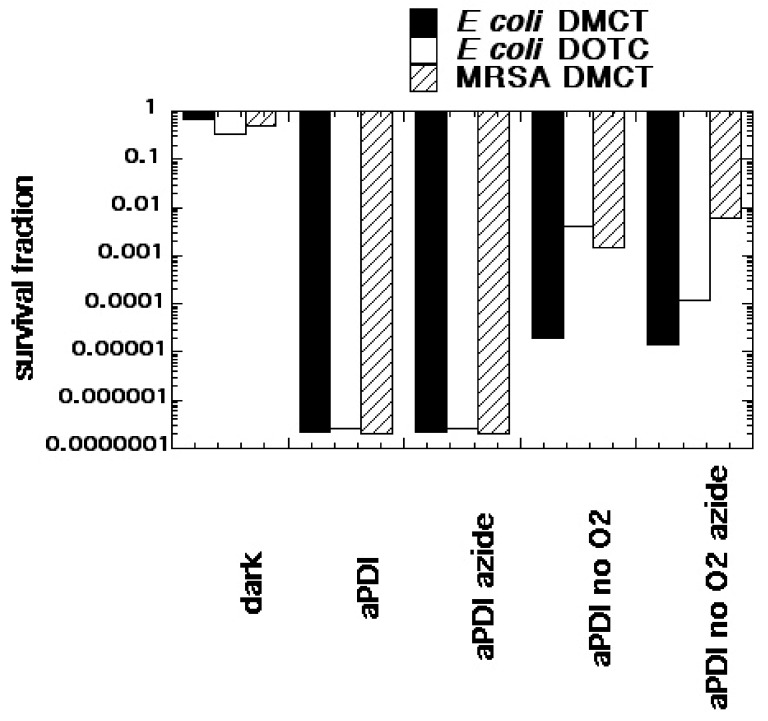
Oxygen-independent bacterial photoinactivation mediated by tetracyclines. Bacterial cells (*E. coli* or MRSA 10(8) CFU/mL) were incubated with tetracyclines (DOTC or DMCT, 100 µM) for 30 min with or without addition of sodium azide (50 mM), and either in air or bubbled with N_2_/Ar. At the end of incubation 10 J/cm^2^ of UVA (DOTC) or blue (415 ± 15 nm) light (DMCT) was delivered. Values are calculated based on no treatment control. Reproduced from [51] (open access).

We formed the hypothesis that tetracyclines accumulated in bacterial ribosomes, where they could be photoactivated with blue/UVA light producing microbial killing via ROS generation. Moreover, it was also possible that photoactivated covalent cross-links could be formed between tetracycline molecules and ribosomal proteins, in a similar manner to the covalent cross-links formed by photoactivated psoralens. There is some literature evidence for this hypothesis in that radiolabeled tetracyclines were used as a type of photoaffinity labeling to identify the actual ribosomal binding site [52]. We tested this hypothesis using demeclocycline (DMCT) activated by blue light and doxycycline (DOTC) activated by UVA light [51] (see structures in Figure 4). We compared aPDI in an atmosphere of ambient oxygen and of nitrogen, and we used sodium azide as a quencher to delineate the involvement of singlet oxygen. Figure 5 shows that both DOTC + UVA light or DMCT + blue light eradicated (> 6 logs killing) both MRSA and *E. coli.* The bacterial killing was unaffected by addition of sodium azide (50 mM) as a quencher. When the oxygen was replaced by nitrogen, there were still around 2–5 logs of killing remaining. Addition of azide in the presence of nitrogen did not inhibit the killing, but on the contrary actually produced a 1-log increase in the case of *E. coli*, DOTC, and UVA light. It is likely that there are contributions from all three photochemical pathways (Type I—hydroxyl radicals, Type II—singlet oxygen, and Type III—oxygen-independent) in the antibacterial effects of photoactivated tetracyclines [51].

## 8. Inorganic Salts

In recent years the Hamblin laboratory has published several papers concerning the potentiation of aPDI by the addition of simple inorganic salts [15,53]. These salts have included potassium iodide [54], potassium bromide [55], sodium azide [56], potassium thiocyanate [57], potassium selenocyanate [58], and sodium nitrite [59]. We have investigated the mechanisms of this potentiation in some depth, and it is quite a complex question when all the different salts are considered [15]. The most powerful and versatile salt is potassium iodide. With iodide the mechanism often involves the intermediacy of singlet oxygen, especially with PS known to largely operate via Type II photochemical pathways. These predominantly Type II PS include, Photofrin [60], Rose Bengal [61], anionic and cationic porphyrins [62], amongst others. 

The overall reaction between iodide anion and singlet oxygen produces triodide anion and hydrogen peroxide as shown in Equation (1). Triodide anion is equivalent to free molecular iodine.

^1^O_2_ + 3I^−^ + 2H_2_O → I_3_^−^ + 2H_2_O_2_ (1)

It is proposed that the mechanism first involves the addition of singlet oxygen to iodide anion to produce peroxyiodide anion [63] (Equation (2)).

^1^O_2_ + I^−^ → IOO^−^ (2)

Peroxyiodide is then protonated to produce iodine hydroperoxide (Equation (3)), which then adds a second iodide anion to produce HOOI_2_^−^ (Equation (4)).

IOO^−^ + H^+^→ HOOI (3)

IOOH + I^−^ → HOOI_2_^−^ (4)

The unstable intermediate HOOI_2_^−^ could decompose via several pathways, but it is suggested that one pathway produces iodine and hydrogen peroxide (Equation (5)), while another pathway produces a pair of reactive radicals (Equation (6))

HOOI_2_^−^ + H^+^ → I_2_ + H_2_O_2_ (5)

HOOI_2_^−^ → I_2_•^−^ + HOO• (6)

Pathway (5) accounts for the production of long-lived antibacterial species (i.e., I_2_ + H_2_O_2_) that can continue to kill microbial cells long after the light is switched off [53]. It should be noted that there are some reports that a combination of iodine and hydrogen peroxide exert a synergistic antibacterial effect compared to either species tested alone [64].

The interesting question is, what happens when aPDI is carried out in the presence of added iodide, but in the absence of oxygen, thus removing the possibility of singlet oxygen mediated reactions? The answer appears to be that what happens depends on the type of PS and how easily it can accept an electron (or in other words how easily it is reduced). 

**Figure 6:**
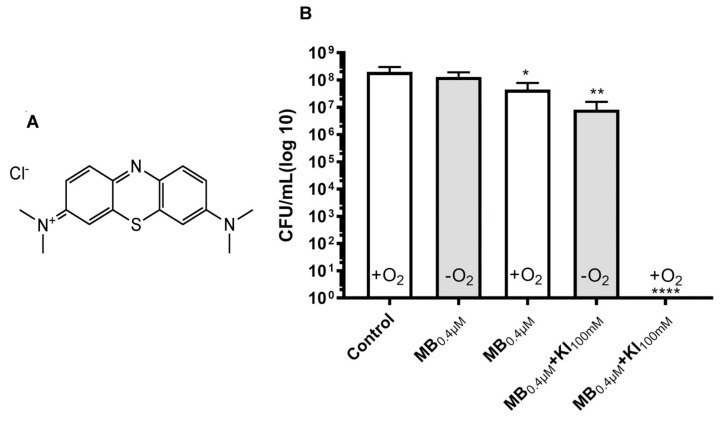
Oxygen-independent bacterial killing mediated by MB plus potassium iodide. (**A**) Structure of MB. (**B**) Antimicrobial effect of MB (0.4 μM) with or without KI (100 mM) plus 6 J/cm^2^ 660 nm light against *E. faecalis* planktonic cells in presence and absence of oxygen (anaerobic incubator). Reproduced with permission from [69]; Copyright Elsevier.

Methylene blue (MB) is a phenothiazinium salt, which has been widely employed to mediate aPDI in vitro [65], in vivo [66], and in human clinical studies [65] (see Figure 6 for structure). MB was one of the first PS whose antibacterial activity we reported was potentiated by addition of potassium iodide (KI) [54]. MB is considered to have a significant contribution of the Type I photochemical mechanism in its aPDI activity, especially when compared to porphyrins [67]. We also reported that the aPDI activity of intravesicular MB plus red light was potentiated by addition of KI in a female rat model of bladder infection caused by bioluminescent uropathogenic *E. coli* [68].

In a recent study we investigated the potentiation of MB-mediated aPDI by addition of KI [69]. We used *Enterococcus faecalis* and studied planktonic cells in vitro, and biofilms growing in 96-well plates and on dentin sections from human teeth. aPDI using a low concentration of MB (0.4 µM) plus 660 nm light (6 J/cm^2^) killed only about 1 log in the presence of oxygen (Figure 6). When oxygen was removed in an anaerobic chamber, there was no killing at all. When the aPDI was repeated with the addition of KI (100 mM) and the presence of oxygen, there was complete killing (~8 logs). When the oxygen was removed in the presence of KI, there were still 2 logs of killing remaining. We propose that the excited state of MB can undergo a 1-electron transfer to form the MB radical cation and the iodine radical anion as shown in Equation (7). These two highly reactive radicals could damage the bacterial cells.

MB* + 2I^-^→ MB•^+^ + I_2_•^−^ (7)

**Figure 7:**
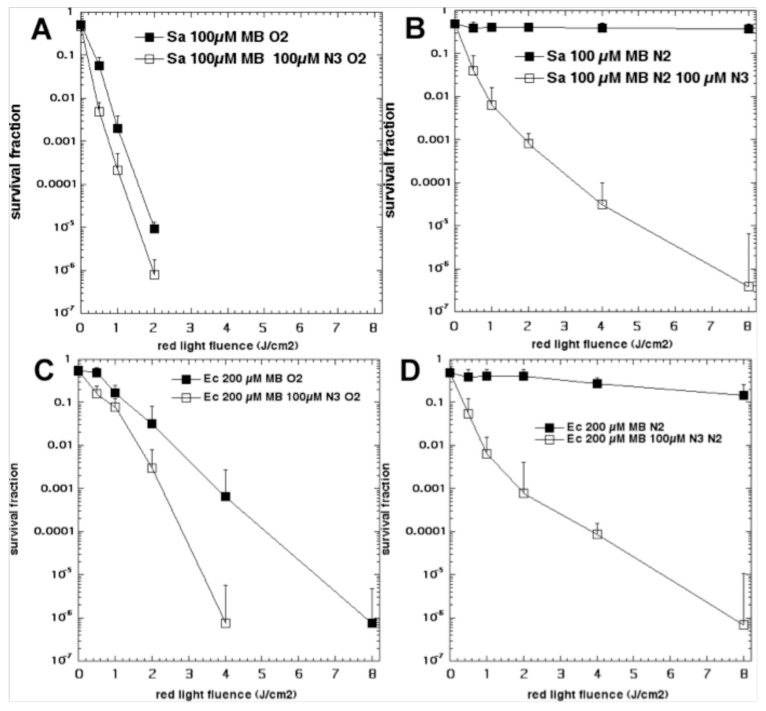
**Oxygen-independent bacterial killing mediated by MB plus sodium azide.** Bacteria (10(8) cells/mL) were incubated with MB for 30 min and then with addition or not of NaN3 (100 µM), followed by removal or not of oxygen by bubbling with nitrogen, and illumination with up to 8 J/cm^2^ of 660-nm light. (**A**) *S. aureus* and 100 µM MB in oxygen; (**B**) *S. aureus* and 100 µM MB in nitrogen; (**C**) *E. coli* and 200 µM MB in oxygen; (**D**) *E. coli* and 200 µM MB in nitrogen. Reproduced with permission from [56]; Copyright Elsevier.

We have also shown that aPDI mediated by MB could be potentiated by addition of sodium azide [56]. Initially we expected that azide could quench singlet oxygen and therefore inhibit bacterial killing, as has been reported for other PS [70]. However, we discovered that instead of the bacterial killing being inhibited, it was “paradoxically potentiated”. We compared the killing of *S. aureus* and *E.coli* mediated by MB and 660 nm light in the presence and absence of azide and in the presence and absence of oxygen [56]. Figure 7 shows the results. For *S. aureus* in the presence of oxygen, MB at 100 µM gave a light-dose dependent loss of CFUs reaching eradication at > 2 J/cm^2^ of 660 nm light. Addition of azide (100 µM) gave approximately one log of extra killing. When the experiment was repeated in the absence of oxygen (nitrogen/argon), the killing without azide was almost abolished (< 1 log). However, in the presence of azide and absence of oxygen, the bacterial killing remained pronounced with eradication obtained at 8 J/cm^2^. When the experiments were repeated with *E. coli*, similar results were obtained. In the presence of oxygen, MB at 200 µM led to 6 logs killing at 8 J/cm^2^, while when azide (100 µM) was added 6 logs of killing was obtained at 4 J/cm^2^, and overall 1–3 extra logs of killing was achieved. When the atmosphere was changed to nitrogen/argon the killing without azide was almost abolished, while in the presence of azide, 6 logs of killing was obtained at 8 J/cm^2.^

Experiments were carried out to elucidate the mechanism [56]. Spin-trapping using 5,5-dimethyl-1-pyrroline-N-oxide (DMPO) plus electron spin resonance identified the formation of superoxide radicals in the presence of oxygen and the absence of azide, but this switched to azide radicals identified by the characteristic spin-splitting pattern when azide was added. The formation of azide radicals was also seen in the presence of azide and the absence of oxygen. Therefore, we proposed that a 1-electron transfer occurred between the excited MB and the azide anion to form a pair of radicals, as shown in Equation (8).

MB* + N_3_^-^→ MB•^+^ + N_3_• (8)

We have studied fullerenes as antimicrobial PS for some time [71,72]. Fullerenes are closed cage molecules constructed solely from carbon atoms (60,70 or more) and take the shape of a soccer ball. Fullerenes have a highly conjugated system of double bonds and therefore have significant absorption bands in the visible spectrum (mainly in the blue and UVA regions). The fullerene cage can accept a large number of electrons, up to six, due to its triply-degenerate molecular orbitals that fall somewhere between diamond and graphite [73]. 

**Figure 8:**
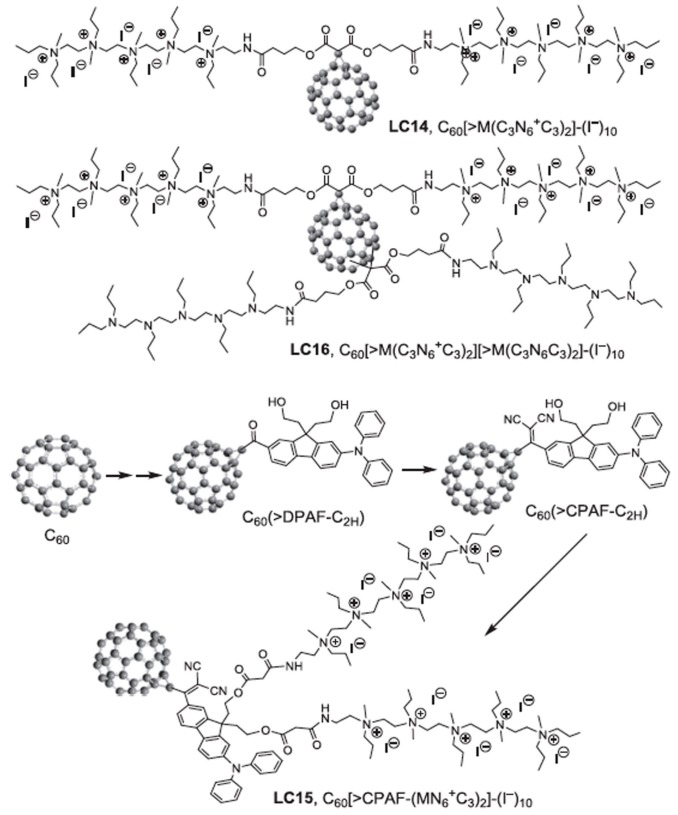
**Structures of polycationic functionalized fullerenes.** LC14 has two chains each of five quaternized nitrogen groups, making it decacationic. LC15 has a similar arrangement of two chains of 5 quaternized nitrogens, but this time attached to a light-harvesting antenna called CPAF. LC16 has two chains of 5 quaternized nitrogens, but in addition has two additional chains of 5 tertiary nitrogens (ten in all).

**Figure 9:**
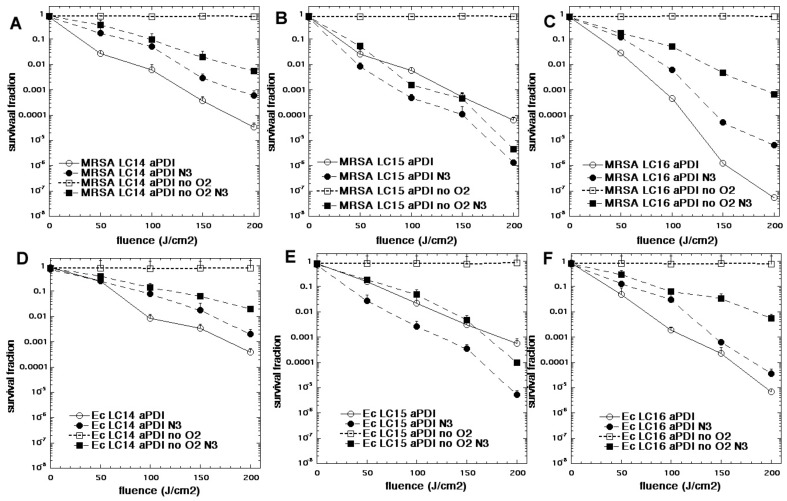
**Oxygen-independent bacterial killing mediated by fullerenes and sodium azide**. Bacteria (10(8) cells/mL) were incubated with fullerene compounds at 10 µM for 1 hour, followed by exposure to increasing fluences of while light (400–700 nm). aPDI was carried in the presence or absence of sodium azide (10 mM) and in the presence or absence of oxygen (75% N_2_/25% Ar). (**A**) MRSA + LC14; (**B**) MRSA + LC15; (**C**) MRSA + LC16; (**D**) *E. coli* + LC14; (**E**) *E. coli* + LC15; (**F**) *E. coli* + LC16. Adapted from data in [74].

In one study we compared three functionalized fullerenes with attached polycationic chains to make the compounds water-soluble and enabled them to bind to bacteria [74]. These compounds were called LC14, LC15, LC16 (Figure 8). LC14 had two chains each of five quaternized nitrogen groups, making it decacationic. LC15 had a similar arrangement of two chains of 5 quaternized nitrogens, but this time attached to a light-harvesting antenna called CPAF (1,1-dicyanoethylenyl})-9,9-dialkyl-2-diphenylaminofluorene). LC16 also possessed two chains of 5 quaternized nitrogens, but in addition had two additional chains of 5 tertiary nitrogens (ten in all) that were designed to act as electron donors. All three fullerenes were compared as PS (10 µM) when activated by broad-band white light to kill MRSA and *E. coli* in the presence and absence of azide (10 mM) and in the presence and absence of oxygen [74]. Figure 9 shows the results. In general, the activity of the three fullerenes was in the order LC16 > LC15 > LC14. As expected, the Gram-positive MRSA was more susceptible to killing than the Gram-negative *E.coli.* In the absence of azide, the removal of oxygen completely abolished the aPDI activity. In the presence of azide and oxygen, there was still killing remaining in the case of LC14 and LC16 (although it was reduced). However, in the case of LC15 addition of azide increased the killing of both MRSA and *E. coli.* In the absence of oxygen, the addition of azide substantially restored the killing, and in the case of LC15 brought it back to the levels found with oxygen present. Although the formation of azide radicals was not specifically tested for in this study, by analogy with the MB data shown above, it can be hypothesized that the fullerene molecules accept photoinduced electron transfers from the azide anion thus producing azide radicals. Moreover, each fullerene molecule could theoretically produce six individual azide radicals, as opposed to just one from MB.

## 9. Discussion and Conclusions

The present review has identified three different instances where oxygen-independent antimicrobial photoinactivation has been demonstrated. Although the studies that have so far been conducted have concentrated on bacteria, the technique could in principal be applied to viruses and fungi. Psoralens in particular would be expected to allow photoinactivation of viruses in the absence of oxygen. Two of the cases described, psoralens and tetracyclines, rely on specific binding between the PS compound and a defined molecular structure inside the microbial cells. In the case of psoralens this binding molecule is the double helix structure of DNA [75] or RNA [76], and in the case of tetracyclines, this structure is one or more proteins of the 30S ribosomal subunit [77]. These PS that bind to their targets can then be activated in situ using light of the correct wavelength, which in both cases is fairly short wavelength light (UVA or blue) that possesses sufficient energy to carry out photochemical reactions involving the breakage or forming of covalent bonds. Covalent bonds are then formed between the PS molecule itself and the adjacent microbial structure, which can be termed the production of “photoadducts”. Because these reactions are intrinsically oxygen-independent, it is not surprising that the bacterial killing is still effective in the absence of oxygen, or even in the case of psoralens, is actually more effective. 

In the third example, potentiation of aPDI by addition of inorganic salts, we propose that the mechanism is fundamentally different. While many of the mechanisms that operate with inorganic salts clearly do depend on the presence of oxygen. In this case singlet oxygen can react with the salt anion to produce bactericidal moieties, whether stable or short-lived. However, in at least the two examples discussed above, the mechanism is oxygen-independent. We propose that the inorganic anions can transfer an electron to the excited state of the PS (probably the triplet state), thus forming the PS radical anion and the inorganic radical. We think that the inorganic radicals are more likely to be the species responsible for attacking the microbial cells, but we cannot rule out that the PS^•–^ could also be an antimicrobial reactive species. The fact that azide radicals have been demonstrated using the ESR spin-trapping technique in the case of MB plus red light provides support for this assertion [56]. If the electron transfer mechanism operates between the excited state PS and the salt anion, then its likelihood will depend on the relative redox potentials for oxidation of the salt and for reduction of the PS. Moreover, molecules like fullerenes can have as many as six separate redox potentials, each one for the successive addition of an electron into the delocalized electron cage.

It will be asked whether oxygen-independent aPDI will ever have any clinical applications. In the case of psoralens, thousands of individuals have already been treated with psoralen and UVA light. In some cases the patients’ skin has been treated for dermatological disorders, but in other cases they have received platelets, plasma, and other blood products that have been treated ex vivo for antimicrobial sterilization. Moreover, extracorporeal photopheresis has employed PUVA with marked success for diverse hematological disorders. However, we cannot trace any reports of PUVA being used to treat an actual bacterial infection (never mind an anaerobic infection), even in animal models of wound or burn infections. It might be objected that PUVA has been shown to cause skin cancer in some individuals when repeated excessively, but its use for a clinical aPDI application would be expected to be limited in the number of repetitions. Tetracyclines have now been used as antibiotics for almost eighty years, but so far (and somewhat surprisingly) there have been no attempts to activate tetracyclines (either systemically or topically administered) using light in order to improve the treatment of localized infections. Perhaps the evidence that tetracyclines also work as PS in the absence of oxygen may break this logjam. When it comes to traditional PS potentiated by addition of inorganic salts, we believe that aPDI potentiated by KI will undergo clinical testing in the foreseeable future. However, the additional activity provided by KI is not particularly high in the absence of oxygen. The effectiveness of aPDI potentiation by added salts in the absence of oxygen is much higher in the case of azide, but the toxicity of azide salts will likely preclude any clinical testing. Moreover, it is not yet entirely clear which types of anaerobic or hypoxic infections in humans would benefit from effective oxygen-independent aPDI, although endodontic sterilization in dental root canals is a possible candidate [69].
